# Exploring Subpopulations for Epidemiological Precision Nutrition Research: The Example of *Phenylalanine Hydroxylase (PAH)* Genetic Variation

**DOI:** 10.3390/nu18111811

**Published:** 2026-06-04

**Authors:** Anoushka Dhawan, Sophia M. Khan, Madison L. Fennell, Clara E. Cho, Jennifer M. Monk, Justine R. Keathley

**Affiliations:** Department of Human Health Sciences, College of Biological Sciences, University of Guelph, Guelph, ON N1G 2W1, Canada; adhawa01@uoguelph.ca (A.D.); skhan57@uoguelph.ca (S.M.K.); mfenne05@uoguelph.ca (M.L.F.); claracho@uoguelph.ca (C.E.C.); jmonk02@uoguelph.ca (J.M.M.)

**Keywords:** phenylketonuria, PKU, heterozygotes, PKU parents, executive functioning

## Abstract

**Background/Objectives:** Biological factors such as genetics contribute to nutrition-related outcomes, but nutritional epidemiological studies often lack consideration of genetics despite evidence of their functional impacts on health and cognition. *Phenylalanine hydroxylase (PAH)* genetic variation has been hypothesized to influence health and cognitive outcomes due to evidence of metabolic perturbations in *L*-phenylalanine to *L*-tyrosine hydroxylation, including plausible downstream effects on catecholamine neurotransmitters among not only individuals with phenylketonuria (PKU) [homozygotes for *PAH* mutations] but also PKU carriers [heterozygotes]. Related to these metabolic perturbations, diminished executive functioning has been observed in individuals with PKU, even when treated, but research is lacking exploring this outcome in PKU carriers. The present study aims to detail methods for stratifying populations based on genetic variation, for use in epidemiological precision nutrition research. It further provides an exploratory exemplar of such research through population stratification by *PAH* genetic variation (i.e., PKU carriers vs. non-carriers), while providing the first descriptive data on executive functioning skills using the validated Executive Skills Questionnaire—Revised (ESQ-R) tool with *PAH*-genetically stratified groups (PKU carriers and non-carriers). **Methods:** Participants were ≥18 years of age and *PAH* heterozygotes (PKU carriers) or non-carriers. Levels of executive functioning were self-reported anonymously online and included the validated Executive Skills Questionnaire—Revised (ESQ-R) tool. Data were analyzed using t-tests, chi-square tests, ANOVAs, and ANCOVAs. **Results:** Respondents (n = 99, n = 79 carriers and n = 20 non-carriers) consisted of males (22.2%) and females (77.8%), primarily of European ancestry. There were no significant differences between groups (carriers vs. non-carriers) for total scores (mean ± SD ESQ-R score carriers = 17.41 ± 14.01; non-carriers = 14.95 ± 10.00), but carriers scored significantly worse than non-carriers for the ESQ-R item “I have trouble making a plan” in the adjusted model. **Conclusions:** This study provides a methodological exemplar for exploring genetically stratified subpopulations in epidemiological precision nutrition research.

## 1. Introduction

Nutritional epidemiological research has historically failed to consider genetic variation, which can be important for hypothesis generation, population stratification, and interpretation of results, among other factors. It is well known that genetic variation influences metabolic processes, and it has often been observed that the stratification of populations based on genetic variation can lead to striking differences in health responses to diet. For example, Vallée Marcotte et al. were able to clearly discriminate between “responders” and “non-responders” to omega-3 supplementation for triglyceride lowering by stratifying participants based on genetic variation [1]. In addition, genetic variation can influence caffeine metabolism and related clinical outcomes. Studies have demonstrated that upon stratifying the population based on *CYP1A2* genetic variation (i.e., “fast” vs. “slow” caffeine metabolizers), when caffeine intake exceeds the equivalent of approximately 2–3 cups of coffee per day, “slow metabolizers” of caffeine are at a significantly higher risk of cardiovascular and renal disease compared to “fast metabolizers” [2,3]. Precision nutrition can be defined as dietary recommendations that consider individual profiling, such as metabolomic, genomic, proteomic, and metagenomic data, in order to optimize health status [4]. Stratifying populations by genetic variation and other elements of precision nutrition can help improve methodological rigor in nutritional epidemiology; this concept has been highlighted in a recent report from the National Institutes of Health’s workshop on precision nutrition [5]. For example, genetic variation influencing metabolism should be used to generate hypotheses, with the aim of helping to improve our understanding of specific population subgroups that may experience unique health risks in response to nutrition interventions [6]. Furthermore, stratifying populations based on genetic variations can improve our understanding of discrepant results in epidemiological research. As a further example, some intervention research has found that *L*-tyrosine (Tyr) supplementation can improve cognitive outcomes (ex. working memory) while others have found no effect [7]. However, when researchers stratified participants based on genetic variation, differences in *DRD2* genotypes impacted variability in responses to Tyr supplementation [8]. While research consistently considers confounding factors such as demographic variables, genetic variation has not commonly been considered as one of these factors.

With stratifying populations based on genetic variation, it is also important to generate descriptive data within genetically stratified subgroups. Grimes and Schulz note that “descriptive studies often represent the first scientific toe in the water in new areas of inquiry” [9]. They play a critical role in characterizing the distribution of health-related variables and providing foundational knowledge about specific populations and conditions [9]. Importantly, descriptive studies serve to inform hypotheses for future research that aim to draw inferences from the data, causal or otherwise [9].

Phenylketonuria (PKU) is the most common genetically inherited metabolic disease [10,11]. The condition is caused by mutations in the *phenylalanine hydroxylase (PAH)* gene, which encodes the PAH enzyme [11]. Mutations in the *PAH* gene lead to reduced or absent PAH enzymatic activity, significantly impairing the conversion of the amino acid *L*-phenylalanine (Phe) to Tyr [11]. Elevated circulating levels of Phe can arise when PKU is not well controlled, for example, when not following a strict diet low in Phe-containing foods, without pharmaceutical treatment, or when left untreated altogether [11]. High Phe levels can lead to neurological damage, intellectual and developmental disabilities, and mental illnesses [11]. In addition, excess Phe can competitively inhibit the L-type amino acid transporter 1 (LAT1) at the blood–brain barrier, limiting the transport of all other large neutral amino acids (LNAAs), including Tyr and tryptophan, to the brain and potentially leading to deficiencies in neurotransmitter production [12]. PKU treatment is individualized, typically including dietary restriction of protein and aspartame, which contain high amounts of Phe, while supplementing with Phe-free foods and formulas consisting of Tyr and other amino acids [13]. Pharmacological treatments include sapropterin, a synthetic co-factor for the PAH enzyme, which is effective in a subset of responsive patients, pegvaliase, an enzyme-substitution therapy, and others [14,15]. Current guidelines recommend maintaining circulating Phe levels ≤ 360 μmol/L in individuals with PKU, as lower circulating Phe levels are associated with higher IQ, a recommendation that is supported by a high certainty of evidence [14].

PKU follows an autosomal recessive inheritance pattern; thus, biological parents or children of PKU patients are heterozygotes for a single *PAH* variant. While PKU is a rare genetic condition affecting approximately 1 in 10,000 individuals, a notable 2% of the population (1 in 50) are *PAH* heterozygotes (i.e., PKU carriers) [16]. The higher prevalence of PKU carriers within the population is highly relevant, as evidence suggests that PKU carriers exhibit an intermediate subclinical metabolic phenotype for PKU. For example, Phe-loading trials have demonstrated that carriers of PKU have higher blood levels of Phe and lower levels of Tyr compared to non-carrier controls, which demonstrates reduced capacity for Phe-to-Tyr conversion [17,18,19,20,21]. However, these trials lack the inclusion of clinical outcomes and genetic sequencing, highlighting the need for complementary methods that explore clinical outcomes such as executive functioning [17,18,19,20,21]. Studies using liver biopsies have also supported the reduced metabolic efficiency of the PAH enzymatic pathway in PKU carriers compared to non-carriers [22,23]. Whether these metabolic disruptions translate into clinical outcomes, such as altered cognitive executive functioning, has yet to be thoroughly explored. Currently, PKU carriers are generally considered to be clinically “unaffected.” However, early research has suggested that carriers may exhibit lower IQ and worse cognitive functioning capabilities compared to non-carrier controls [24,25], but these outcomes have yet to be robustly investigated. 

Executive functioning is a high-order cognitive skillset that refers to the ability to control, organize, and prioritize time and activities [26]. It can be sub-categorized into *plan management*, *time management*, *organization*, *emotional regulation* and *behavioral regulation* [27]. In patients with PKU, impairments in executive functioning are commonly noted; metabolically, these likely stem from disturbances in monoamine neurotransmitters, including deficiencies in serotonin, norepinephrine, and dopamine, which directly relate back to PKU impairments in the PAH metabolic pathway (i.e., reduced endogenous Tyr production) [11,28,29,30,31]. In 2018, a study demonstrated that parents of PKU children (PKU carriers) performed worse on cognitive tasks than non-carriers [25]. Specifically, they reported that carriers had worse executive functioning, delayed and immediate memory, and processing speed when compared to non-carrier controls [25]. While PKU carriers tend to exhibit disruptions in the production of PAH pathway metabolites, such as Phe and Tyr [17,18,19,20,21], further studies exploring clinical outcomes, including executive functioning in this population, are needed. Descriptive, reference data on executive functioning in this population is also needed. Biological sex is also important to consider in this work, given that it appears to play a role in executive functioning [32]. Specifically, structural and functional differences have been observed in the male and female brain, and moreover, males and females have been shown to differ in how they manage household tasks, discipline, and emotional support, which are directly related to executive functioning [33,34].

Overall, many factors have been demonstrated to impact executive functioning. One factor being parenthood that generally has a protective effect on levels of executive functioning, and parents’ executive functioning capabilities are also predictors of their children’s executive functioning capabilities [35,36,37]. Among biological parents of patients affected by PKU (who are also *PAH* heterozygotes/genetic carriers for PKU), only one small study has explored executive functioning in this population, and the results were intriguing [25]. This study compared PKU carrier parents of adult PKU patients (n = 12) to non-carrier controls (n = 14) and observed overall lower executive functioning scores among the parents (carriers) compared to controls. More specifically, carriers scored significantly worse than non-carrier controls on tests related to processing speed and executive functioning, such as set-shifting abilities and inhibitory control [25]. Given the abovementioned differences in PAH pathway metabolism among PKU carriers, executive functioning outcomes could be related to higher serum Phe following protein intake, and may be an important outcome to understand among the 1 in 50 individuals who are genetic carriers for PKU [16].

To provide a strong foundation for future epidemiological precision nutrition research, it is important to develop descriptive data on priority nutrition-related outcomes of interest for specific subsets of the population based on their unique metabolic, genetic and/or health statuses. While genetic sequencing and metabolomic profiling are not typically part of routine clinical care, there is movement towards integrating these multi-omics technologies into practice settings [38]. As such, precision nutrition research is needed to inform the use of genetic and metabolic information clinically.

Given the metabolic perturbations observed in PKU carriers alongside early evidence of possible impacts on executive functioning (plausibly related to Phe [protein] intake, which is typically high in a standard, non-PKU diet), the present study aims to provide preliminary descriptive data on executive functioning skills among genetic carriers and non-carriers of PKU. This study also aims to detail a methodological exemplar for stratifying populations based on genetic variation, to inform epidemiological precision nutrition research. The exemplar included herein focuses on population stratification by *PAH* genetic variation and is exploratory at this time; as such, the results should be interpreted with caution. Between-group comparisons intend to provide foundational descriptive data that can help inform future epidemiological precision nutrition studies (e.g., for use in sample size calculations). The comparison explicitly accounts for precision-related factors, such as biological sex, genetic variation, metabolic differences and other variables relevant to precision nutrition. Notably, this is the first study to report on executive functioning skills among PKU carriers using the validated Executive Skills Questionnaire—Revised (ESQ-R) tool [19]. This descriptive data can be used to inform future epidemiological precision nutrition research that aims to compare carriers to non-carriers, while inferring effects of carrier status on executive function.

## 2. Materials and Methods

### 2.1. Study Conceptualization and Hypothesis Generation

An epidemiological precision nutrition approach was utilized to design the present study. The study hypothesis was developed based on previous literature, which consistently indicates that there are metabolic differences in Phe metabolism among carriers of PKU compared to non-carriers [17,18,19,20,21]. Thus, with dietary Phe intake (i.e., from a standard protein-containing diet), carriers experience greater increases in Phe levels compared to non-carriers, which could plausibly be neurotoxic. In addition, carriers exhibit reductions in Tyr compared to non-carriers, which could plausibly influence neurocognitive outcomes due to the impact on downstream metabolites, including catecholamine neurotransmitters [18]. Therefore, in carriers who are currently presumed to be unaffected and thus do not follow a diet restricted in Phe, these metabolic differences could plausibly influence clinical outcomes, including executive functioning.

Given the abovementioned subclinical *PAH* pathway disturbances among PKU carriers, it was hypothesized that there could be clinical manifestations (for example, differences in executive functioning) in this genetically stratified subpopulation, representative of an intermediate phenotype of PKU compared to non-carriers. Thus, executive functioning was selected as the outcome of interest given that executive functioning is commonly impaired in PKU [11,28,29,30,31], and *PAH* genetic variation was selected as the precision nutrition group stratification factor.

### 2.2. Recruitment and Participants

Participants were recruited through advertisements from clinics, organizations, and social media pages related to PKU, metabolic conditions, and/or genomics (Supplementary Table S1). All participants in the present study were 18 years of age or older and confirmed genetic carriers or non-carriers of PKU. Descriptive characteristics of participants were all self-reported (e.g., self-identified ethnicity, self-reported income, etc.). All participants provided their written informed consent. This study was reviewed and approved by the University of Guelph Research Ethics Board (REB#23-02-009).

### 2.3. Executive Skills Questionnaire—Revised (ESQ-R)

Levels of executive functioning were anonymously self-reported by participants using Qualtrics survey software (XM Platform) and evaluated using the validated ESQ-R tool [39]. The ESQ-R comprises 25 questions that are scored on a 4-point Likert scale, indicating: never or rarely [0 points], sometimes [1 point], often [2 points], or very often [3 points]. While the complete ESQ-R can be scored to obtain an indicator of overall executive functioning, the following sub-categories can also be individually calculated and scored to determine different domains of executive functioning capabilities including plan management [score based on 11 questions], time management [score based on 4 questions], organization [score based on 3 questions], emotional regulation [score based on 3 questions] and behavioral regulation [score based on 4 questions]. The ESQ-R provides an estimate of executive function based on participant self-report, rather than direct behavioral assessment. Higher scores are indicative of worse executive functioning skills.

### 2.4. Data Analysis

Data were analyzed using SPSS version 29.0.2 and analyses included descriptive statistics, as well as analysis of variance (ANOVAs), analysis of covariance (ANCOVAs), t-tests, and chi-square/Fisher’s exact tests (for categorical data) to compare outcomes between *PAH*-genetically stratified groups. In cases where expected counts were <5 in the chi-square analyses, Fisher’s exact statistics were used. A *p*-value < 0.05 was indicative of statistical significance. When scoring the total ESQ-R and executive functioning sub-categories, missing data was handled by assuming a zero score for cases where participants missed responding to only a single question; scores were then calculated accordingly. In cases where a participant missed responding to more than one question required to calculate the score, their data were removed from the total ESQ-R and sub-category analyses. ANCOVAs were used for the adjusted models to control for parent status (i.e., being a parent to a child with PKU). Using the descriptive data obtained from this study, a sample size calculation was also conducted using the comparison of two means, as described by Seabrook 2025 [40].

## 3. Results

### 3.1. Participants

Participants (n = 99) were primarily female (77.8%), of European ancestry (88.8%) and above the median annual household income in the US [41] with a mix of parents who had a younger child with PKU (<18 years of age; 61.9%) and those who had an adult child with PKU (≥18 years of age; 38.1%). In the full sample, 63.6% of participants had a child/children with PKU, primarily among the carriers, as expected. Other than parent status (i.e., being a parent of a child/children with PKU), there were no significant differences in participant demographic characteristics between groups. Descriptive characteristics of participants are further described in Table 1. Groups were also comparable for current or past medical history of anxiety, depression, and/or ADHD (Table 1).

### 3.2. Sample Size Calculation

A sample size calculation was conducted from the present dataset in order to inform sample sizes for adequately powered future research in this area. Using the comparison of two means as described by Seabrook 2025 [40], with a minimally important difference of 5 points in total ESQ-R score and an estimated standard deviation (SD) of 13 derived from the present sample (pooled SD), we determined that n = 212 participants (n = 106 carriers and n = 106 non-carriers) would be required to detect significant differences between groups, with an alpha of 0.05 and power of 80%.

### 3.3. Executive Functioning

Overall ESQ-R scores were relatively low in the present sample (carriers: 17.41 ± 14.01; non-carriers: 14.95 ± 10.00). After stratifying participants based on the possession of either one *PAH* genetic variant (carriers/heterozygotes) or none (non-carriers), there were no significant differences between groups (carriers vs. non-carriers) for total ESQ-R scores in this exploratory analysis (Table 2). Minimal data imputation was used for missing values (see Table 2 footnote).

There were also no significant differences between groups for any of the sub-categories (*plan management, time management, organization*, and *emotional regulation*) in the unadjusted analyses (Table 2). These results did not appreciably change in the adjusted model. Among the descriptive scores for sub-categories (means and SD), carriers had overall higher scores in all categories compared to non-carriers (though not significantly higher) except *behavioral regulation* in which both groups had identical scores, with the highest (i.e., worst) scores in the sub-category *organization*, and the lowest (i.e., best) in the sub-category *plan management*.

There were significant differences between groups for the individual question “I have trouble making a plan”, in which carriers had significantly higher (worse) scores than non-carriers in the main adjusted model (Table 2).

## 4. Discussion

The principal aim of the present study was to provide a methodological exemplar for conducting epidemiological precision nutrition research. The example provided included the formation of a nutrition-related hypothesis based on previously published data [18,19,20,21] demonstrating metabolic differences between genotype groups. Descriptive data is reported, which was used to inform a sample size calculation for a larger, adequately powered study. While the present study is the largest study to date exploring descriptive executive functioning scores in PKU carriers compared to non-carriers, it was still underpowered to detect significant differences. Nonetheless, the establishment of reference scores for this *PAH* heterozygote subpopulation can help strengthen the design and interpretation of future precision health research related to executive function and PKU carriers [21].

For example, the descriptive data can be used to inform sample size calculations for future research. We determined, using the methods described by Seabrook 2025 [40], that a sample size of 212 participants would be required to detect significant differences between groups. Ensuring an adequate sample size is important given that underpowered samples are prone to Type II error and unstable effect estimates. This helps to ensure that findings are interpreted appropriately without over- or underestimating effects or generating undue concern among PKU carriers. We reiterate that our analyses are descriptive and exploratory in nature and intend to provide preliminary reference data, serving as a foundation to guide the methodological design of future studies. The small sample size limits statistical power and our ability to detect significant differences between groups. The imbalance in groups is another limitation to the robustness of the comparative analyses. The current study did not evaluate dietary Phe intake to evaluate if this would impact executive functioning in carriers differently than non-carriers. However, presumably, both carriers and non-carriers were not following a severely Phe-restricted diet. Future research should include larger, controlled samples to enable robust comparisons between carriers and non-carriers. Future research should also evaluate dietary protein (Phe) intake to evaluate if higher vs. lower protein diets influence clinical outcomes in *PAH* heterozygotes. Patients with mild hyperphenylalaninemia, a mild presentation of PAH deficiency, encompass another important subpopulation for future research [33,34].

In the present study, PKU carriers demonstrated relatively low ESQ-R scores, reflective of generally strong executive functioning capabilities. This is particularly notable given that most carriers were also parents of a child with PKU, and there are notable cognitive and emotional demands of caring for a child with this condition [42,43]. This highlights these parents’ impressive adaptability and resilience in the face of complex, lifelong caretaking responsibilities. The generally high executive functioning capabilities that we observed are consistent with other research exploring executive functioning among parents in general, which indicates high executive functioning skills overall [36]. However, even after adjustment for parent status, results remained unchanged. The ESQ-R has a minimum possible score of zero (indicative of high executive functioning) and a maximum possible score of 75 (indicative of poor executive functioning). Participants in the present study scored an average of 17.41 (carriers) and 14.95 (non-carriers), suggesting that they typically responded with “never or rarely” or “sometimes” to the items in the questionnaire, indicating a relatively high degree of executive functioning. In practice, the ESQ-R is often used to compare changes from baseline to follow-up within the same patient, and a 5-point change in the total ESQ-R score is considered a “clinically meaningful” change [39]. When comparing scores from the present population to others that have been previously reported in the literature, we found that scores were comparable [44]. For example, among working adults in Malaysia, the mean total reported ESQ-R scores were 20.54 ± 10.3 compared to the present study where non-carriers reported mean scores of 14.95 ± 10.00, and carriers reported 17.41 ± 14.01 [34]. Future research should implement the use of the ESQ-R assessment in non-carriers of PKU to compare various population means of executive functioning.

Interestingly, we noted a relatively high variability in the data for the ESQ-R total scores, especially in the carriers (SD = 14.01). This variability may reflect multiple unmeasured factors, and one possible explanation that warrants further investigation is heterogeneity in the severity of genetic variants among the PKU heterozygotes, whereby carriers with variants associated with classical PKU (and thus lower PAH enzymatic activity) [45] may have worse executive functioning than those with variants associated with mild PKU or mild hyperphenylalaninemia. However, this remains speculative, as genotype-specific data were not available in the present study. Our results also demonstrate that while overall ESQ-R scores did not differ significantly between groups, carriers had significantly higher scores than non-carriers for the question “I have trouble making a plan,” which falls within the sub-category *plan management*. However, there were no significant between-group differences for the sub-category *plan management* scores. Further, there may be an association with the extent of caregiver burden that a parent may be experiencing, as individuals with classical PKU tend to have more severe dietary restriction and symptoms than those with a milder version of PKU [11], thus complicating the nature versus nurture debate. Future research is needed to explore this further. Moreover, it is important to note that while we observed no significant differences, reporting null findings is still important as it strengthens the scope of literature on a topic and avoids bias in the body of knowledge [21]. Further epidemiological data for this subpopulation related to PKU-associated clinical outcomes, such as executive functioning and other neurocognitive outcomes, is necessary to strengthen the body of evidence.

It has been previously documented that PKU carrier status is directly related to PAH enzymatic function [22,23]. Therefore, the production of downstream metabolites of this pathway that are relevant to psychological and cognitive outcomes, such as dopamine, serotonin and norepinephrine, may also be impacted in carriers [20,25]. Future precision nutritional epidemiological research should explore this. In addition, broader dietary factors such as omega-3 fatty acid intake have been associated with executive functioning in the general population [46] and may represent additional variables worth considering in future studies. However, none of these biological or nutritional pathways were directly measured in the present study, and therefore, they should be interpreted as background hypotheses rather than explanatory mechanisms for the observed findings. Lastly, sex differences should also be considered in future epidemiological studies as executive functioning and metabolism could both differ in females compared to males.

While the study was designed with a precision nutrition approach, with a hypothesis generated based on previously reported metabolic differences depending on *PAH* genetic variation, a significant difference in executive functioning was not observed. The null findings observed in the present analyses may be due to the relatively small sample size or due to a true lack of effect; regardless, reporting null findings remains critical to reduce publication bias. It is important to reiterate that the principal aim of the present study was not to compare carriers to non-carriers; rather, our goal was primarily to provide a methodological exemplar for epidemiological precision nutrition, while providing descriptive, reference data on executive functioning in PKU carriers and non-carriers. Future adequately powered studies will be necessary to confirm or refute any observed trends and null findings and to explore potential differences between carriers and non-carriers. Establishing reference data for populations that could plausibly differ from the general population is important for guiding precision nutrition research and practice. Future work should expand reference datasets beyond PKU carriers to other genetically or otherwise stratified groups. For example, a subpopulation of PKU (e.g., mild hyperphenylalaninemia vs. classical PKU) may have a differing risk of symptoms (i.e., executive function), highlighting the importance of reference data for clinicians and researchers to base disease management and future studies on. Moreover, executive functioning represents only one potential clinical outcome, and there are numerous cognitive and health outcomes, such as working memory, mental health, and mood, that warrant investigation in PKU carriers in future research [47]. In addition, future work in the field of precision nutrition aimed at assessing the practicality of genetic sequencing and metabolic profiling in research and clinical practice is needed. It is also important to note that the sample in the present study consisted primarily of individuals of European ancestry, which limits generalizability to other populations. The potential of response bias may also limit the study results. Participants were aware of their PKU carrier status while completing the survey, which could influence self-reported (ESQ-R) questionnaire responses. In addition, a substantial proportion of PKU carriers (78.5%) in this study were parents of children with PKU, which may introduce additional unmeasured confounding related to caregiving burden, stress, sleep disruption, and other psychosocial factors that are independently associated with executive functioning. This makes it challenging to distinguish the potential genetic effects from caregiving-related influence. Although parent status was adjusted for in the analyses, residual confounding from these related factors may still be present. Beyond caregiver burden, there are other factors that also impact executive functioning in the general population, such as sleep [48]. A larger adequately powered study, which includes data on a number of possible confounders, is needed to account for these factors. Furthermore, while the ESQ-R is a validated measure, it is still self-reported in nature, and it does not directly assess behavioral executive function. The responses to the questionnaire may be influenced by subjective perception as well as the time of day, day of the week, or season in which participants completed the questionnaire. It is also important to note that since executive functioning was assessed using a single validated tool (ESQ-R), it is possible that other self-reported questionnaires or objective executive functioning tasks could yield different results. Future research incorporating direct behavioral measures of executive functioning could provide complementary and more objective data to strengthen conclusions in this field. Collectively, these limitations restrict the ability to draw strong inferences regarding group differences and reinforce the exploratory and descriptive nature of the study.

Overall, in the present study, the first descriptive, reference ESQ-R scores were established for the subpopulation of PKU carriers. This is an important first step as we work towards greater knowledge of precision nutrition in this niche subpopulation.

## 5. Conclusions

The study provides a methodological exemplar in which a population was genetically stratified, based on a hypothesis that was generated from existing metabolic data. The study also describes the first set of reference executive functioning scores among genetic carriers of PKU, using the validated ESQ-R tool. Further precision nutrition epidemiological research comparing PKU carriers to non-carriers, as well as exploring other outcomes such as mental health and metabolic perturbations in this population, will be a critical future research avenue in order to better characterize PKU carriers and the nature-nurture pathophysiology of possible clinical observations.

## Figures and Tables

**Table 1:** Descriptive characteristics of participants (PKU carriers [*PAH* heterozygotes] vs. non-carriers).

Characteristics (Number of Participants, Frequency; or Mean ± SD)	PKU Carriers [*PAH* Heterozygotes] (n = 79)	PKU Non-Carriers (n = 20)	*p*-Value	PKU Carriers and Non-Carriers (Combined, n = 99)
Sex	Male: n = 18, 22.8% Female: n = 61, 77.2%	Male: n = 4, 20.0% Female: n = 16, 80.0%	0.789	Male: n = 22, 22.2% Female: n = 77, 77.8%
Ethnicity	Black: n = 1, 1.3% East Asian: n = 1, 1.3% Latin American: n = 1, 1.3% Oceania: n = 1, 1.3% South Asian: n = 1, 1.3% European: n = 70, 88.6% Other: n = 4, 5.1%	European: n = 17, 89.5% Other: n = 2, 10.5%	0.924	Black: n = 1, 1.0% Chinese: n = 1, 1.0% Latino: n = 1, 1.0% Oceania: n = 1, 1.0% South Asian: n = 1, 1.0% European: n = 87, 88.8% Other: n = 6, 6.1%
Annual household income (USD)	165,007 ± 267,482	112,675 ± 97,378	0.419	154,054 ± 242,385
Has a child/children with PKU	Yes: n = 62, 78.5% No: n = 17, 21.5%	Yes: n = 1, 5.0% No: n = 19, 95.0%	<0.001 *	Yes: n = 63, 63.6% No: n = 36, 36.4%
Has a child/children with PKU < 18 years of age	n = 38, 61.3%	n = 1, 100.0%	0.429	n = 39, 61.9%
Has a child/children with PKU ≥ 18 years of age	n = 24, 38.7%	n = 0, 0.00%	0.429	n = 24, 38.1%
Current or past medical history of one or more of the following: anxiety, depression, ADHD	n = 32, 40.5%	n = 8, 40.0%	0.967	n = 40, 40.4%

USD: United States Dollars; PKU: phenylketonuria; ADHD: Attention Deficit Hyperactivity Disorder. * Indicates statistical significance at *p* < 0.05.

**Table 2:** Results of the ESQ-R among PKU carriers [*PAH* heterozygotes] and non-carriers.

ESQ-R Item Score (Mean ± SD)	PKU Carriers [*PAH* Heterozygotes] (n = 79)	PKU Non-Carriers (n = 20)	Unadjusted *p*-Value	Adjusted *p*-Value
Individual scores for Questions 1–25				
I act on impulse	0.54 ± 0.66	0.50 ± 0.51	0.780	0.777
I say things without thinking	0.51 ± 0.68	0.65 ± 0.49	0.375	0.752
I lose things	0.95 ± 0.86	0.80 ± 0.77	0.481	0.625
I have a short fuse	0.61 ± 0.74	0.55 ± 0.69	0.754	0.940
I get upset when things don’t go as planned	1.00 ± 0.66	0.65 ± 0.59	0.033 *	0.534
I run out of steam before finishing a task	0.68 ± 0.84	0.45 ± 0.60	0.246	0.497
It’s hard for me to set priorities when I have a lot of things to do	0.71 ± 0.86	0.55 ± 0.69	0.448	0.617
My desk or workspace is a mess	0.87 ± 0.99	0.85 ± 0.93	0.924	0.559
I have trouble keeping my house or room clean	0.85. ± 1.05	0.70 ± 0.92	0.566	0.895
I have trouble estimating how long it will take to complete a task	0.67 ± 0.90	0.70 ± 0.73	0.894	0.545
I’m slow at getting ready for school, work, or appointments	0.51 ± 0.95	0.50 ± 0.69	0.978	0.998
If the first solution to a problem doesn’t work, I have trouble thinking of a different one	0.49 ± 0.75	0.30 ± 0.47	0.143	0.462
I skip checking my work for mistakes, even when the stakes are high	0.42 ± 0.74	0.25 ± 0.44	0.189	0.420
I get annoyed when tasks are too hard	0.68 ± 0.74	0.55 ± 0.60	0.459	0.571
It’s hard for me to put aside fun 16. activities to start things I know I need to do	0.72 ± 0.86	0.55 ± 0.60	0.404	0.749
I have trouble with tasks where I know I need to come up with my own ideas	0.61 ± 0.72	0.55 ± 0.76	0.754	0.713
It’s hard for me to tell how well I’m doing on a task	0.53 ± 0.71	0.50 ± 0.69	0.859	0.834
I have trouble reaching long-term goals	0.58 ± 0.79	0.65 ± 0.75	0.731	0.599
I “go with my gut” when making decisions	1.08 ± 0.78	1.00 ± 0.32	0.499	0.763
I get so wrapped up in what I’m doing that I forget about other things I need to do	0.84 ± 0.95	0.55 ± 0.60	0.206	0.379
Little things frustrate me	0.82 ± 0.69	0.80 ± 0.70	0.896	0.685
I have trouble getting back on track if I’m interrupted	0.82 ± 0.90	0.50 ± 0.61	0.134	0.202
I have trouble making a plan	0.42 ± 0.67	0.20 ± 0.41	0.057	0.046 *
I miss the big picture	0.54 ± 0.68	0.30 ± 0.47	0.055	0.092
I live for the moment	0.91 ± 0.79	0.90 ± 0.55	0.952	0.767
Sub-Category and Overall Scores				
Plan Management ^3^	0.59 ± 0.60	0.44 ± 0.45	0.281	0.434
Time Management ^4^	0.68 ± 0.77	0.58 ± 0.52	0.552	0.856
Organization	0.89 ± 0.86	0.78 ± 0.70	0.611	0.903
Emotional Regulation	0.81 ± 0.55	0.67 ± 0.55	0.304	0.918
Behavioral Regulation ^2^	0.76 ± 0.53	0.76 ± 0.33	0.974	0.860
TOTAL ESQ-R SCORE ^1^	17.41 ± 14.01	14.95 ± 10.00	0.472	0.748

* Indicates statistical significance at *p* < 0.05. ^1^. A zero value was imputed for three participants who had missing values for one question; two participants had missing values for >1 question and were removed from the analysis (therefore n = 97). ^2^. A zero value was imputed for one participant who had a missing value for one question. ^3^. A zero value was imputed for two participants who had missing values for one question; one participant had missing values for >1 question and was removed from the analysis (therefore n = 98). ^4^. One participant had missing values for >1 question and was removed from the analysis (therefore n = 98).

## Data Availability

Data is available upon reasonable request.

## Supplementary Materials

The following supporting information can be downloaded at: https://www.mdpi.com/article/10.3390/nu18111811/s1, Table S1: Recruitment support channels.
