# Supplementary material for: Exploring Subpopulations for Epidemiological Precision Nutrition Research: The Example of Phenylalanine Hydroxylase (PAH) Genetic Variation
Source: Nutrients. 2026 Jun 4;18(11):1811. doi: 10.3390/nu18111811 (PMC13259265; doi:10.3390/nu18111811)
Supplement: Supplementary file 1 [file nutrients-18-01811-s001.zip › nutrients-4318010-supplementary.pdf]

SUPPLEMENTARY FILE

Supplementary Table S1: Recruitment Support Channels

Name	Link
CanPKU+: Canadian PKU & Allied Disorders Inc.	https://www.canpku.org/
E.S.PKU: European Society for Phenylketonuria and Allied Disorders Treated as Phenylketonuria	https://www.espku.org/
California Coalition for PKU & Allied Disorders	https://www.ccpkuad.org/
GMDI: Genetic Metabolic Dieticians International	https://gmdi.org/
Facebook Page: 23 and Me forums	https://www.facebook.com/groups/23andmeforums/
Facebook Page: Ancestry-23&me-FTDNA-GEDmatch-DNA-andMore Open Discussion	https://www.facebook.com/groups/opengen
Facebook Page: PKU in Canada: Educate the educators initiative	https://www.facebook.com/groups/200506730079050
Facebook Page: PKU Awareness	https://www.facebook.com/groups/1068139769877119
Twitter Account: Justine Keathley	https://x.com/JustineKeathley/
